# Older adults process the probability of winning sooner but weigh it less during lottery decisions

**DOI:** 10.1038/s41598-022-15432-y

**Published:** 2022-07-05

**Authors:** Hsiang-Yu Chen, Gaia Lombardi, Shu-Chen Li, Todd A. Hare

**Affiliations:** 1 Chair of Lifespan Developmental Neuroscience, Faculty of Psychology, Technische Universität Dresden, Dresden, Germany; 2 Chair of Methods of Psychology and Cognitive Modelling, Faculty of Psychology, Technische Universität Dresden, Dresden, Germany; 3 Department of Economics, Zurich Center for Neuroeconomics, University of Zurich, 8006 Zurich, Switzerland; 4 Centre for Tactile Internet With Human-in-the-Loop (CeTI), Technische Universität Dresden, Dresden, Germany; 5 Neuroscience Center Zurich, University of Zurich and ETH Zürich, 8006 Zurich, Switzerland

**Keywords:** Human behaviour, Computational science

## Abstract

Empirical evidence has shown that visually enhancing the saliency of reward probabilities can ease the cognitive demands of value comparisons and improve value-based decisions in old age. In the present study, we used a time-varying drift diffusion model that includes starting time parameters to better understand (1) how increasing the saliency of reward probabilities may affect the dynamics of value-based decision-making and (2) how these effects may interact with age. We examined choices made by younger and older adults in a mixed lottery choice task. On a subset of trials, we used a color-coding scheme to highlight the saliency of reward probabilities, which served as a decision-aid. The results showed that, in control trials, older adults started to consider probability relative to magnitude information sooner than younger adults, but that their evidence accumulation processes were less sensitive to reward probabilities than that of younger adults. This may indicate a noisier and more stochastic information accumulation process during value-based decisions in old age. The decision-aid increased the influence of probability information on evidence accumulation rates in both age groups, but did not alter the relative timing of accumulation for probability versus magnitude in either group.

## Introduction

Decision-making is an integral part of our daily lives. Goal-directed decisions are those in which we spend time to explicitly evaluate and compare the attractiveness between different choice options^1^. For example, when booking a hotel room people usually consider several features of the room such as its size, hotel location, Wi-Fi availability, and other general facilities. When making a goal-directed choice, the decision-maker may evaluate and compare the features, also called attributes, of different choice options in order to select the option with maximal outcome benefits from their perspective. The characteristics of the decision-maker (e.g., financial status, individual preferences, or age) may affect the valuation and value comparison processes underlying the eventual choices^2–5^.

During healthy aging, structural^6^, functional^7,8^, and neurochemical^9–11^ changes in the fronto-striatal circuitry are well established. This circuitry is also known to underlie value-based decision-making^12,13^; thus, age-related alterations therein may affect older adults’ decision processes^14^. Specifically, several studies showed that when the choices were framed as mixed outcomes of gains and losses, older adults tended to accept more gambles than younger adults even when the probability of winning was low^4,5,15,16^. This tendency to choose gambles with low or even negative expected values (EV)—defined as the multiplicative product of reward probability and magnitude^2,3^—in older adults may indicate a compromised valuation and comparison process. Furthermore, neuroimaging studies on value-based decision-making in old age have revealed an age-related decline in value assessments and representations^14,17–19^. Compared to younger adults, older adults exhibited lower blood-oxygen-level-dependent (BOLD) signal changes that were associated with positive or negative EVs in the ventral striatum^17,19^ or in the anterior insula^18^, respectively.

Although empirical evidence has revealed that the valuation process changes with advancing age, it has also been shown that older adults’ decision-making can be improved by environmental support, specifically by enhancing the saliency of the outcome probability or integrated information about the EV of the choice options. Previous studies have applied a color-coding scheme (e.g., green and red indicating information associated with gains or losses, respectively) as a decision-aid to increase the saliency of EVs^20^ or the outcome probability^21^. Results from these prior studies showed that the salient visual decision-aid improved older adults’ choice behaviors and allowed them to behave more similar to younger adults in making more rational choices. However, questions about how age and information saliency of outcome probability may affect the dynamics of the decision-making process (e.g., when different features of an option start to affect the decision process) are still open. To address these questions, the present study fit a time-varying drift diffusion model^22–24^ to younger and older adults’ choice data under conditions with or without decision-aid from our prior study^21^, in order to investigate the effects of age and enhancing information saliency about outcome probabilities on the relative weightings and temporal dynamics of two defining attributes of valuation (i.e., reward probability and magnitude that frame the EV in the given choice trial). Of note, valuation computation involving multiplicative integration of reward magnitude and probability as proposed in classical decision theoires^2,3^ may be implemented in processes of accumulating different evidence for the decision at different times^22–24^.

Specifically, we used a time-varying drift diffusion model that focueses on the process level during value-based decisions by introducing starting time parameters (henceforth starting time DDM, stDDM). The starting time parameters denote how much earlier (or quicker) one attribute begins to affect the valuation process (i.e., evidence accumulation) relative to the other(s)^23^. Separate considerations of the starting time parameters allow the stDDM to unravel the psychometrics and dynamics of different choice attributes in influencing value-based decision-making, and how these processes may differ between individuals or age groups as well as be affected by the task contexts or demands.

We also investigated whether the amount of thus far accumulated reward affected the current decision process in addition to reward probabilities and magnitudes, which are the constituents of the EV for each choice trial. Participants in this study were paid based on the total of the accumulated reward and the feedback about this amount was given after every decision. Therefore, we developed four versions of the stDDM to test (1) if feedback about the accumulated reward influenced the decision process on the next trial, and (2) when the current trial characteristics (reward probability and magnitude) began to influence the decision process relative to the feedback about past outcomes (accumulated reward).

Using the starting time DDM (stDDM), we investigated how strongly (weighting strength) and when (starting time) the different attributes of choice options influenced decision-making in younger and older adults in conditions with and without the decision-aid. Drift diffusion models are commonly applied to quantify age-related differences in subprocesses of cognitive decisions. For instance, a recent meta-analytic study^25^ reported differences between younger and older adults in three main parameters of a standard drift diffusion model (i.e., one that does not allow for differences in starting times across attributes) that map onto partially distinct processes of perceptual, lexical, and memory-based decisions. In particular, relative to younger adults, older adults showed higher boundary separation (i.e. response caution) and longer non-decision times in lexical decision^26,27^ and memory recognition^26,28^ tasks, as well as slower drift rates in brightness discrimination and memory tasks^25,29^. Notably, the differences between younger and older adults in boundary and non-decision times were consistent across task type and difficulty, while differences in drift rates depended on both task type and difficulty. In light of these previous findings, we hypothesized that older adults would show higher boundary separation and longer non-decision times than younger adults. We also predicted that, compared to younger adults, older adults would show slower drift rates (i.e., smaller weighting strength parameters in the stDDM), potentially indicating age-related increases in the noise of neural information processing^30,31^ that undermines evidence accumulation during value-based decision-making^14,32,33^. Furthermore, we expected that increasing the saliency of the outcome probability would increase the weighting strength of probability information and facilitate the starting times for considering probabilities relative to other attribute information such as reward magnitudes.

## Results

### EV sensitivity on the choice patterns by age and condition

**Figure 1:**
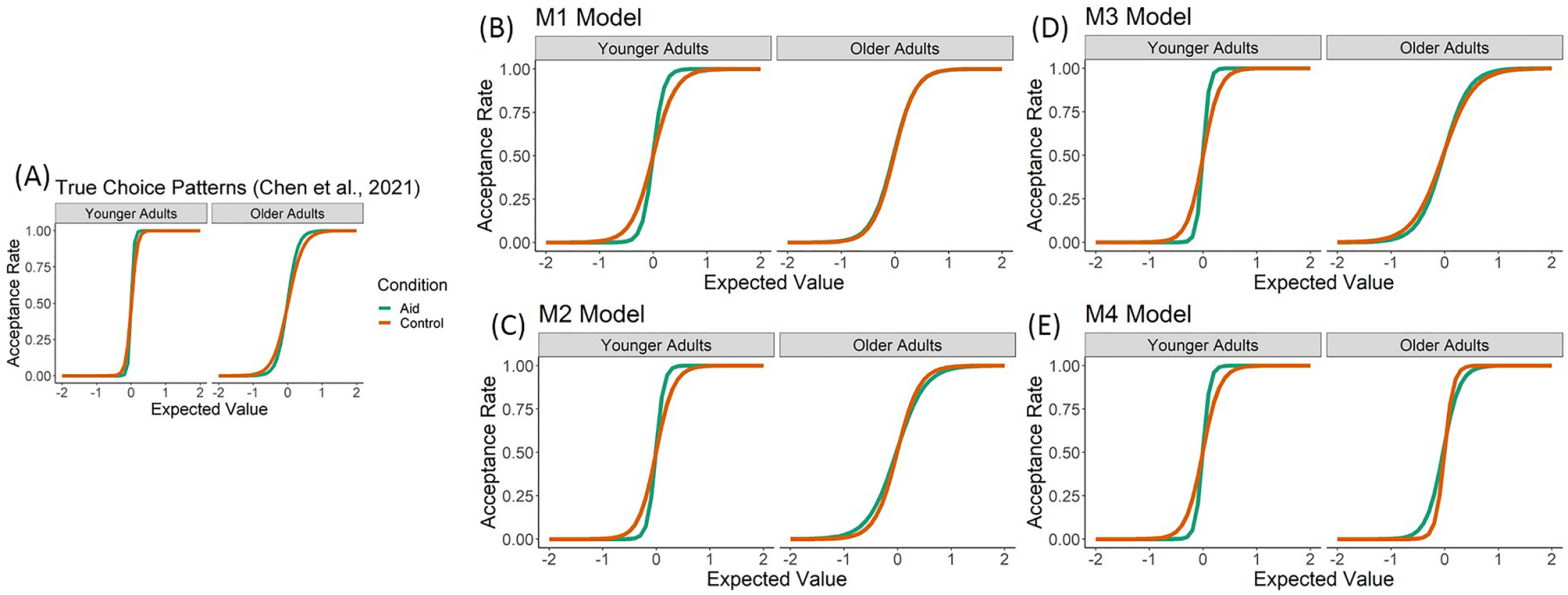
Logistic regression slopes (value sensitivity) for the empirical and simulated data sets. (**A**) The plot shows the empirical choice curves for younger and older adults by conditions [green indicating decision-aid condition and orange indicating control condition; adapted from Chen et al. (2021)]. Relative to younger adults, older adults showed shallower slopes near the point where the gamble’s expected value equals zero, indicating less sensitivity to expected value when making decisions. The decision-aid increased the slopes in both age groups; however, this effect was more pronounced in younger than older adults (Chen et al. 2021). The plots in (**B**–**E**) show the same logistic regression slopes fitted to the simulated choice data produced by the parameters for the four stDDMs that we compared. Only M3 generates the correct pattern for older adults. In older adults, M1 does not generate a difference between decision-aid and control trials, while M2 and M4 generate choices with less value sensitivity in decision-aid than control trials, which is the opposite of the empirical results.

Figure 1A shows the patterns of behavior in decision-aid and control trials for each age group as reported in the original publication from these data^21^. We briefly reiterate those results here for convenience. Value sensitivity is captured by the slope of the logistic regression model that defines EV as multiplicative combinations of reward magnitude and probability (see the Methods section for details). Effects of age groups (younger/older) and experimental conditions (decision-aid/non-aid) on the empirical choice data with a 2 × 2 non-parametric ANOVA revealed main effects on age and condition (*ps* < 0.05). Younger adults showed a higher slope (i.e., higher EV sensitivity) than older adults across both conditions, and the decision-aid increased the slopes in both age groups. In addition, an interaction for age × condition was also present (*p* < 0.05), showing that increased slope was larger in younger than in older adults. Our goal in the current paper is to determine which stDDM specification best accounts for this observed pattern of behavior.

### Model comparisons

We specified and compared four stDDMs that varied in terms of the ways that the different choice attributes (outcome probabilities, reward magnitudes, and amount of previously accumulated rewards) could influence the decision process (see Table 1 and Methods section for details). The plots in Fig. 1B–E show how well the parameters from each of the four stDDMs (M1, M2, M3, and M4) recreate the patterns of EV sensitivity observed in the empirical data. We computed logistic regressions using the empirical data and simulated data from each stDDM following the same procedures described in our prior publication^21^, and those details and results can be found in the Methods and Supplementary Information sections. The M3 specification of the stDDM is the only one that generates the correct patterns of significant differences across age groups and conditions. Similarly, Table 2 reports the root-mean-squared-error (RMSE) between the true and simulated choice outcomes and the deviance information criterion (DIC) value for each model, which also indicates that M3 is the best model. The RMSE was computed using split-half cross-validation and shows similar performance at the choice level for all four models. The DIC values are based on the combination of choice outcomes and reaction times (RTs) in each age group and condition and indicate that M3 is the best fitting model (smaller DIC indicates a better model).

**Table 1:** Summary of four starting time drift diffusion models.

M1	This model does not include any influence of previously accumulated reward on the current decision
M2	This model tests if previously accumulated reward levels change the starting point bias for evidence accumulation in the current trial
M3	This model tests if previously accumulated reward levels influence the evidence accumulation rate in the current trial and this influence starts at the beginning of the evidence accumulation process
M4	This model tests if previously accumulated reward levels influence the evidence accumulation rate in the current trial and whether this influence starts before or after the information about reward probability and magnitude of the current lottery

**Table 2:** Root-mean-squared-error and deviance information criterion value of each model for the model comparison.

Group and condition	Model							
	M1		M2		M3		M4	
	RMSE	DIC	RMSE	DIC	RMSE	DIC	RMSE	DIC
**YA**								
Control	0.20 ± 0.08	5104.54	0.19 ± 0.08	4991.09	0.19 ± 0.08	4817.21	0.20 ± 0.08	4890.84
Aid	0.17 ± 0.09	1000.20	0.17 ± 0.09	914.21	0.17 ± 0.09	798.73	0.17 ± 0.09	967.99
**OA**								
Control	0.28 ± 0.10	15,002.14	0.28 ± 0.10	14,953.44	0.28 ± 0.10	14,560.64	0.28 ± 0.10	14,613.66
Aid	0.27 ± 0.10	12,978.38	0.27 ± 0.10	12,752.02	0.26 ± 0.10	12,572.21	0.26 ± 0.10	12,620.63

This table reports the mean and standard deviation of RMSE and the DIC value calculated by the hierarchical Bayesian Markov Chain Monte Carlo method for each age group and each condition. Abbreviations: YA = younger adults; OA = older adults; Aid = decision-aid; RMSE = root-mean-squared-error; DIC = deviance information criterion.

Altogether, the model comparisons suggest that the M3 model is the best fitting model and capable of reproducing the choice patterns as what we found in the empirical datasets. Therefore, we report comparisons of parameter estimates between age group and condition based on the M3 stDDM. This model, on one hand, specifies that previously accumulated reward influences the evidence accumulation rate (i.e., drift rate) rather than the starting point bias (as tested in M2), and that the accumulated reward affects the evidence accumulation process from its onset. On the other hand, the M3 model allows for separate starting times for probability and magnitude information into the evidence accumulation process.

### Effects of age group and condition on the stDDM (M3) parameters

**Figure 2:**
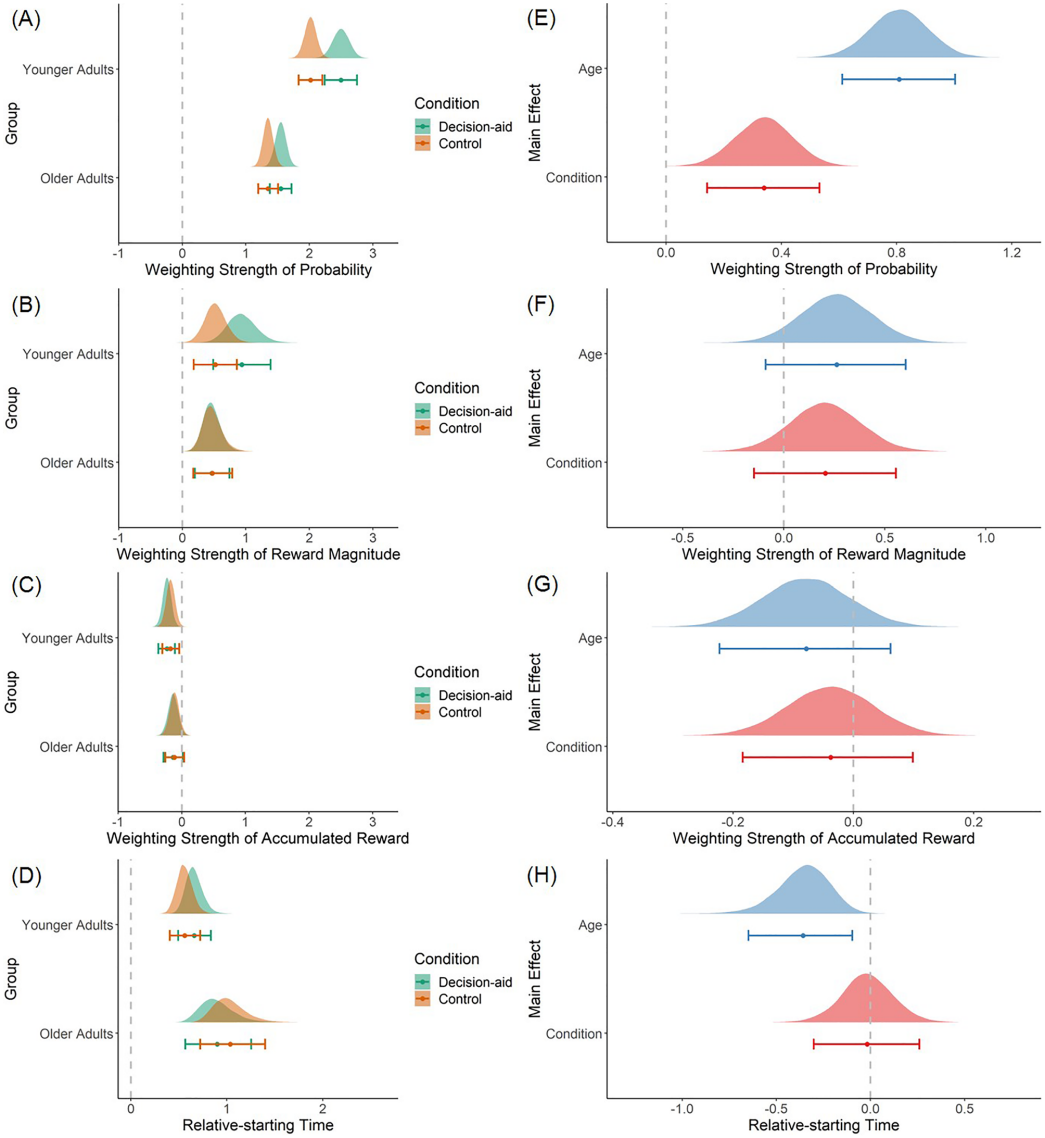
Effects of age group and condition on the drift weight and relative starting time parameters of stDDM (M3). (**A**–**D**) Posterior distributions of the group-level parameters from the best fitting model (M3) in all experimental conditions in both age groups. (**E**–**H**) Age and condition differences of the posterior distributions of the hyperparameters in the best fitting model (M3). Age group differences are shown as younger minus older adults. Condition differences are shown as aid minus control. Note. Dots and error bars shown below the distribution plots indicate the mean and 95% highest density interval, respectively.

**Figure 3:**
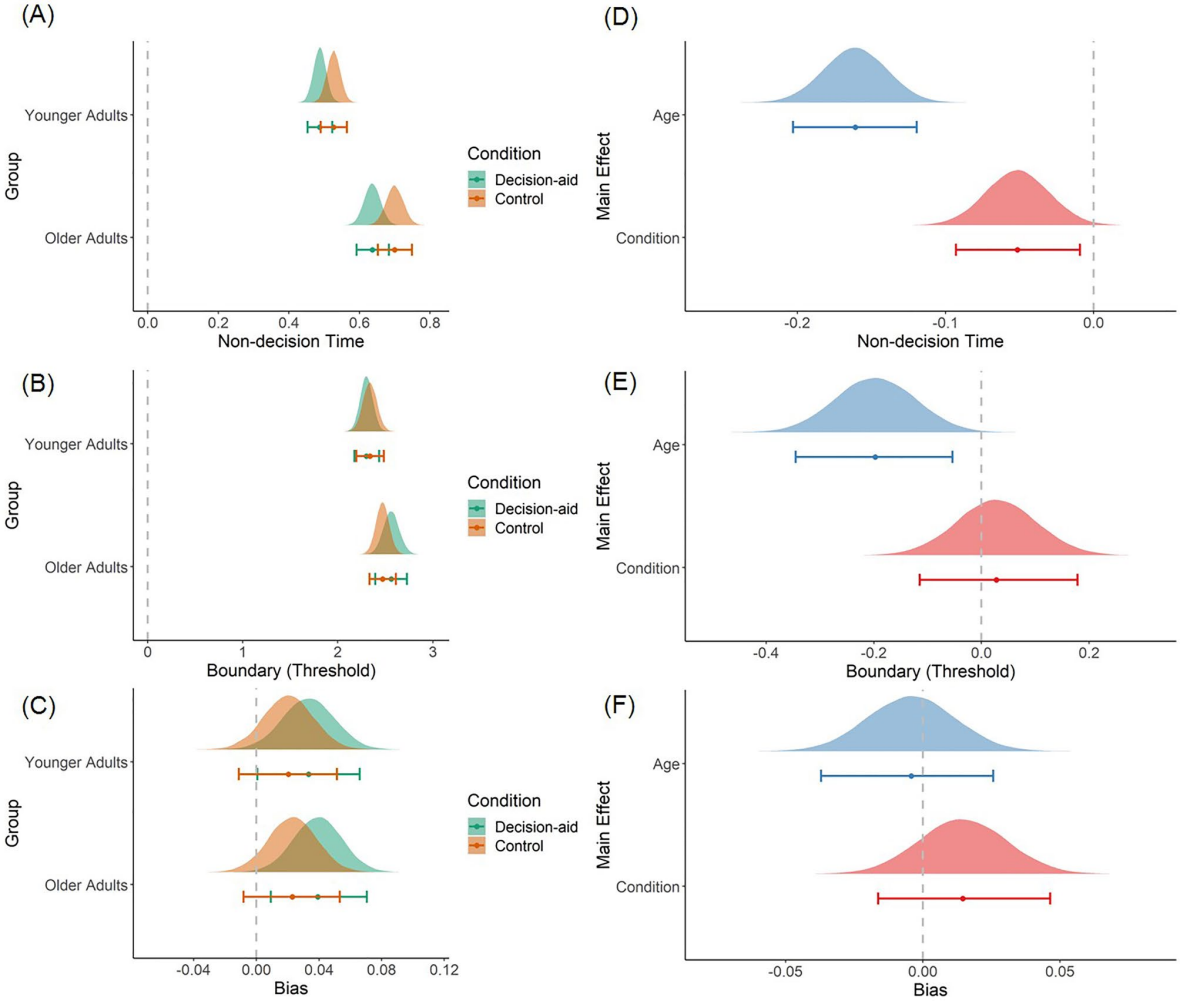
Effects of age group and condition on the non-decision time, boundary separation (threshold), and starting point bias parameters of stDDM (M3). (**A**–**C**) Posterior distributions of the group-level parameters from the best fitting model (M3) in all experimental conditions in both age groups. (**D**–**F**) Age and condition differences of the posterior distributions of the hyperparameters in the best fitting model (M3). Age group differences are shown as younger minus older adults. Condition differences are shown as aid minus control. Note. Dots and error bars shown below the distribution plots indicate the mean and 95% highest density interval, respectively. The distributions of the bias parameter are shifted by the parameter values minus 0.5 to have the same reference point at zero as other parameters.

We found that the influence of probability information on evidence accumulation differed across age groups and conditions. While current reward magnitudes and previously accumulated rewards significantly increased or decreased the probability of taking the gamble, respectively, the influence of those factors on evidence accumulation rates did not differ by age or condition. Table 3 reports the main effects and interactions between age groups and conditions on the stDDM (M3) parameter estimates. Figures 2 and 3 show the posterior distributions for each parameter within each age group and condition (left panels) as well as the differences in the posterior distributions between age groups and conditions (right panels). In addition to the differences in the weighting strength and onset timing of probability information on evidence accumulation (see the next paragraph), older adults had longer non-decision times (Fig. 3A,D) and higher boundary separation (Fig. 3B,E) than younger adults, as predicted based on the previous findings noted in the introduction (Table 3).

**Table 3:** Differences in the posterior distributions for each parameter of the best fitting model (M3) across age groups and conditions.

Difference	Parameter							
	Probability coefficient	Magnitude coefficient	Probability minus magnitude coefficient	Accumulated reward coefficient	Relative-starting time	Non-decision time	Boundary (threshold)	Bias (starting point)
Age (YA–OA)	0.81 [0.61 1.00] 1.00	0.26 [− 0.09 0.60] 0.93	0.55 [0.14 0.93] 1.00	− 0.08 [− 0.22 0.06] 0.14	− 0.36 [− 0.65 − 0.10] 0.00	− 0.16 [− 0.20 − 0.12] 0.00	− 0.20 [− 0.35 − 0.05] 0.00	0.00 [− 0.04 0.03] 0.40
Condition (Aid–Control)	0.34 [0.14 0.53] 1.00	0.21 [− 0.15 0.56] 0.88	0.13 [− 0.26 0.54] 0.75	− 0.04 [− 0.18 0.10] 0.30	− 0.02 [− 0.30 0.26] 0.44	− 0.05 [− 0.09 − 0.01] 0.01	0.03 [− 0.11 0.18] 0.65	0.01 [− 0.02 0.05] 0.82
Age × Condition [(YA: Aid–Control)—(OA: Aid–Control)]	0.28 [− 0.13 0.66] 0.92	0.43 [− 0.27 1.12] 0.89	− 0.14 [− 0.96 0.64] 0.36	− 0.04 [− 0.32 0.24] 0.40	0.24 [− 0.32 0.79] 0.81	0.02 [− 0.06 0.11] 0.71	− 0.13 [− 0.41 0.17] 0.19	0.00 [− 0.07 0.06] 0.46

This table reports the mean and 95% HDI in square brackets of the distribution of the differences in posterior estimates for each stDDM parameter. The posterior probability showing that the difference is greater than zero is listed below the HDI for each comparison. Posterior probabilities equal to 0.00 or 1.00 are due to rounding and do not indicate certainty. Abbreviations: HDI = highest density interval; PP = posterior probability; YA = younger adults; OA = older adults; Aid = decision-aid.

The stDDM allows us to investigate both the weighting strength and relative starting time of each attribute’s influence on the evidence accumulation process during value-based decisions. Both age groups placed more weight on probability than magnitude information (Fig. 2A,B) and began to consider probabilities earlier than magnitudes (Fig. 2D, Table S3). Although the decision processes in both age groups favored probability information over magnitude information in terms of weight and consideration onset times, the extent to which probability was favored with regard to weight and onset times differed by age group. Younger adults placed relatively more decision weight on probability, relative to magnitude, than older adults [mean difference = 0.55, 95% highest density internal (HDI) = [0.14 0.93]; Fig. 2E, Table 3]. On the other hand, older adults began processing probabilities, compared to reward magnitudes, relatively earlier than younger adults (mean difference = − 0.36 s, 95% HDI = [− 0.65 − 0.10]; Fig. 2H, Table 3).

The decision-aid that increased the saliency of probability information changed the drift weight for probability and non-decision time parameters of the stDDM (M3). The weight on probability information increased in the decision-aid relative to control trials (mean difference = 0.34, 95% HDI = [0.14 0.53]; Fig. 2E, Table 3). Non-decision times showed a small but consistent decrease in the aid relative to the control condition (mean difference = − 0.05 s, 95% HDI = [− 0.09 − 0.01]; Fig. 3A,D, Table 3). There were no significant main effects of age or condition on the weight of reward magnitudes (Fig. 2B,F) or starting point bias parameter (Fig. 3C,F). Furthermore, there were also no significant interactions between age group and condition on any of the stDDM parameters. Nevertheless, the aggregate effects of the decision-aid on the stDDM parameters were sufficient to generate the age × condition interactions on EV sensitivity as reported above and shown in Fig. 1.

In addition to the details of the current gamble, previously accumulated reward levels also influenced the decision process. Across all age groups and conditions, the previously accumulated reward had a significantly negative influence on the accumulation rate for evidence in favor of taking the gamble (Fig. 2C, Table S3). In other words, participants were more likely to reject gambles as the previously accumulated reward level increased. There were no differences between age groups and conditions, nor any interaction effects for the influence of accumulated reward on gamble decisions (Fig. 2G, Table 3).

## Discussion

We sought to better understand the effects of enhancing the saliency of probability information on the dynamics of the decision process in older and younger adults. Probability information is an important attribute for value computation, which is difficult for older adults to process^4,33^. Although previous work has shown that a decision-aid that increases the saliency of specific information can shift older adults’ choice behaviors towards normatively optimal strategies (i.e., higher EV sensitivity)^20,21^, how increasing information saliency may affect decisions at the mechanistic level was not well understood. Therefore, we tested four stDDMs to determine how different attribute information (outcome probabilities, reward magnitudes, and previously accumulated rewards) might affect the starting point, drift rate, and timing within the evidence accumulation process quantified by the stDDM.

In addition to the information about the probability and magnitude of gain and loss in the current trial, we tested how cumulative outcomes (e.g., accumulated reward level) may affect individual’s decision-making. Note that although past work comparing older and younger adults’ decisions focused solely on the reward probabilities and magnitudes within the current decision^4,5,20,21^, we found that higher levels of accumulated reward from previous feedback made both older and younger adults less likely to accept further gambles. This finding is consistent with theories suggesting that choices over risks are reference dependent^3,34^. We tested whether this effect occurred because accumulated reward levels changed the starting point bias for the evidence accumulation process (implemented in M2) or the drift rate of evidence accumulation (implemented in M3 and M4).

Given that the information about accumulated reward was provided before the probabilities and magnitude of gain and loss were shown in each trial, it is plausible that accumulated reward levels might create a *prior* bias (i.e. starting point bias) in the decision maker toward or against accepting the current gamble. Alternatively, knowledge of the accumulated reward level might be held in memory during the decision and sampled as evidence for the probability and reward information in the current gamble. The model comparison tests showed that the affect of previously accumulated reward levels on current choices is better explained via an influence on the evidence accumulation rate within the current choice, rather than changes in the initial bias toward or against accepting a gamble. Furthermore, the best fitting stDDM (M3) revealed that previously accumulated reward levels and the probability information in the current choice trial influenced the evidence accumulation process before the magnitude information for all age groups. Although accumulated reward levels had an important impact on decisions for both age groups, the increased saliency of probability information did not change its influence on choices.

At the level of parameters in the best fitting stDDM (M3) that quantify individual components of the decision process, our results are in line with previous studies of decision-making in older and younger adults. Similar to previous reports^25,29^, older relative to younger adults in the present study exhibited higher boundary separation and longer non-decision times in the stDDM. The probability of gain versus loss in the current gamble was the strongest factor in determining decisions for both age groups (Fig. 2). In the decision task we used, the magnitude of the potential gain or loss in each trial was equal, and thus whether the EV of the gamble that was positive or negative was determined by the outcome probability. Therefore, it is not surprising that the probability information had more influence on participants’ decisions than the magnitude information. Although both groups relied more on outcome probabilities to make their decisions, older and younger adults differed in terms of how probability information was favored over magnitude information. Specifically, within the stDDM framework, there are two factors indicating that one attribute information could have more influence on choice outcomes than another: (1) the information could have a higher decision weight and thus a stronger influence on the evidence accumulation slope, or (2) the information could begin to be accumulated earlier in the decision process relative to other types of information. Our results showed that younger adults put more decision weights on probability, relative to reward magnitude, than older adults. On the other hand, older adults showed larger starting time advantages for probabilities than younger adults. The reduced decision weight on probability in older adults may indicate age-related increases in the noise of neural information processing^30,31^. The advanced starting time for probability information in older adults might indicate compensation for the noisier evidence accumulation process to the most relevant attribute (i.e., probability) over the less relevant information (i.e., magnitude). However, it is also possible that slower and nosier information processing in old age may lead to greater asymmetry in accumulation starting times between probability and magnitude because older adults may require more time to incorporate another attribute information into the evidence accumulation process. In other words, older adults may not have solely favored probability over magnitude information as younger adults did, but rather been slower in all cognitive processes, which may prolong the starting time for magnitude information in the accumulation process. Future work comparing older adults’ decisions in tasks in which two or more attributes are equally versus unequally relevant will be needed to address these open questions.

Enhancing the saliency of outcome probabilities led to an increased weighting strength for the probability attribute in both age groups. The amount that probability weights increased, relative to decision weights on the magnitude, did not significantly differ between younger and older adults. None of the individual stDDM parameters showed a significant age group × condition interaction. Nevertheless, the combined changes in stDDM parameters across decision-aid and control trials were sufficient to produce the age group × condition interactions at the group level in EV-sensitivity when we simulated choices using the best-fitting stDDM parameters for each individual and condition. These simulation results highlight the interdependency of the mechanisms underlying decision-making and the utility of fitting generative models to decision behavior to test for effects of interventions on both individual components of the decision process and on the complex synergistic or antagonistic relationships between components that ultimately produce decision behaviors.

In conclusion, beyond existing evidence for adult age differences in the valuation process when decisions are framed as mixed outcomes of gains and losses, our results shed new light on differences between older and younger adults in the dynamics of accumulating evidence about the reward probability and magnitude attributes of the choice options. These differences appear to contribute to the well-established decrease in value-based decision-making in older relative to younger adults. The present findings indicate that the most robust consequence of enhancing the saliency of the outcome probabilities through a color-coded decision-aid is an increase in the weighting strength for probability information during value-based decisions in younger and older adults.

## Methods

### Participants

The behavioral data analyzed here were previously published^21^, but all the stDDM analyses presented here are new. Forty-four younger (18 males; mean age 24.14 ± 3.09 years) and 53 older adults (20 males; mean age 71.85 ± 5.02 years) with normal or corrected-to-normal vision participated in the study, and none of them reported a history of psychiatric or neurological disorders. Because two older adults’ choice performance showed equal choice acceptance rates across all conditions (range 0.4–0.6) regardless of outcome probabilities and reward magnitudes, they were excluded from the analyses. Each participant was paid €15 for two hours of their participation and earned an additional monetary bonus, which was equal to 10% of the total points that the participant obtained in the mixed lottery choice task and converted into Euro cents. Written informed consent in accordance with the Declaration of Helsinki (2008) was obtained from the participants before starting the experiment. Moreover, the study was approved by the ethics committee of Technische Universität Dresden (EK 511112015). For the complete demographic and sample characteristic descriptions, please see^21^.

### Mixed lottery choice task

**Figure 4:**
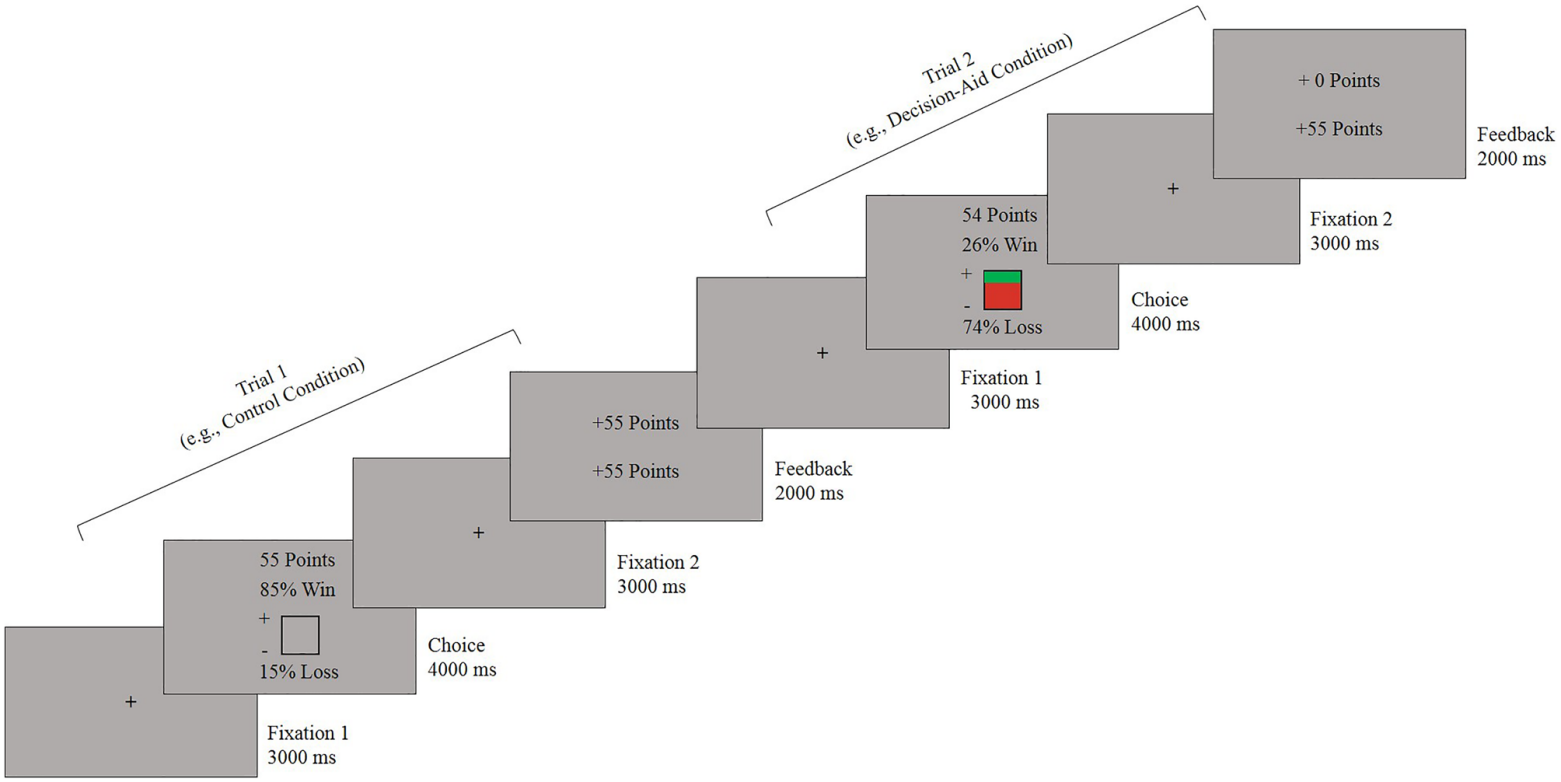
Schematic diagram of the mixed lottery choice task (shown here are examples of a win trial in the 1st trial and the participant accepted the gamble, followed by a loss trial in the 2nd trial, which the participant rejected). On the feedback screen, the top value indicates current gain or loss, whereas the bottom value indicates the accumulated points across trials.

Participants performed a mixed lottery choice task that was developed by Goh et al. (2016). The task included three factors: probability information saliency (using a visually displayed color-coding on the outcome probability), outcome probability, and reward magnitude, and each trial consisted of a choice and a feedback phase. The extended description of the task, stimuli, and trial design is described in Chen et al. (2021). Briefly, the lottery task included win probabilities in the range of 4% to 95% (mean = 50% ± 32%) and reward magnitudes in the range of 2 to 110 points (mean = 56 ± 40). These constituted the EVs [win probability*magnitude + (1 − win probability) * (− magnitude)] in the range of − 94 to 97 (mean = 0 ± 44) of value-based decision-making that was performed either with (decision-aid condition) or without the decision-aid (control condition). During the choice phase, the win and loss probabilities as well as the magnitude of reward points at stake were presented in texts on the screen. To increase probability information saliency, in the decision-aid condition, in addition to being presented in texts, a colored square was shown and within the square, the size of the area denoted by green and red colors represented the win and loss probabilities, respectively. In contrast, in the control condition, the same-sized square was completely filled with the background gray color. Participants had to decide to accept or reject the lottery at stake on each trial of the choice phase. After the choice was made or until a response deadline of 4 s was met, the outcome screen displayed in the feedback phase showed the points gained or lost for the current trial on the upper part of the screen as well as the total accumulated points across trials thus far on the lower part of the screen (see Fig. 4 for trial structure and condition type).

### Starting-time drift diffusion model (stDDM)

Binary choices (accepting vs. rejecting the stake) and RTs from the onset of a given lottery until a choice was made (key press) were recorded. Trials with RTs less than 200 ms or no choice response until the time deadline at 4000 ms were excluded from all analyses (0.57 ± 1.93 trials in younger and 1.63 ± 2.60 trials in older adults were removed). Participants’ binary choices and reaction times were fit with a time-varying DDM^23^. The specific time-varying DDM we used here allows for differences in the time at which each attribute begins to influence the evidence accumulation rate, and thus we refer to it as the starting time DDM (stDDM). We fit the stDDM using the R package Rjags and the JAGS MCMC sampling algorithm^35^. Compared to the standard DDM, which assumes a constant drift-rate within each choice, in the stDDM different attributes (e.g., outcome probabilities, reward magnitudes and previously accumulated rewards) of the choice options can enter into the evidence accumulation process at different time points relative to one another, meaning that the drift-rate can potentially change within a single decision (Eq. 3). Specifically, the model includes a relative-starting time parameter that allows one attribute to start to be considered before another to quantify the temporal effects of multiple attributes in the decision process. The stDDM can be formulated as follows:

1 $$dE\left( t \right) = \mu \left( t \right)dt + \sigma dW\left( t \right) E\left( 0 \right) = E_{0}$$

2 $$\tau = inf\left\{ {t > 0|} \right.E\left( t \right) \notin \left. {\left( { - B,B} \right)} \right\}$$

where $E\left( t \right)$ indicates the evidence accumulation at time *t*, $\mu \left( t \right)$ indicates the drift rate or speed of the accumulation process over time, $\sigma$ indicates the diffusion rate (drift variability), $E_{0}$ indicates the initial bias (starting point), and $\tau$ indicates the reaction time when the accumulated evidence crosses a threshold (boundary) *B*. The drift variability parameter ($\sigma$ in Eq. 1) was fixed as 1 for all our analyses.

In the present study, we developed four models according to four hypotheses (Table 1), and the drift rate, $\mu \left( t \right),$ as well as the starting point bias parameter, *E*_*0*_, are established differently in the four models. In the first (M1) and second (M2) model, we hypothesized that the drift rate parameter depends on the weighted value differences and starting times of the reward probability and magnitude attributes as follows:

3-M1, M2 $$\mu \left( t \right) = \left\{ {\begin{array}{*{20}l} {\omega_{P} V_{P} } \hfill & {if\; s > 0 \wedge 0 < t < s} \hfill \\ {\omega_{M} V_{M} } \hfill & { if\; s < 0 \wedge 0 < t < \left| s \right|} \hfill \\ {\omega_{P} V_{P} + \omega_{M} V_{M} } \hfill & \quad \quad {if\; t > \left| s \right|} \hfill \\ \end{array} } \right.$$

where $\omega_{M}$ and $\omega_{P}$ indicate the weighting strengths (drift coefficients) given to the reward magnitude and probability attribute, respectively, in the current trial. $V_{M}$ and $V_{P}$ are the values of reward magnitude and the difference between win and loss probability at stake, respectively, in the current trial. Note that both reward magnitudes and probabilities were z-scored across all participants and trials before fitting the models. Thus, $\omega_{M}$ and $\omega_{P}$ indicate the effect of a change of 1 standard deviation in each attribute on the drift rate. The relative onset time parameter, ${\text{s}},$ indicates the time step at which the reward probability attribute enters the accumulation process relative to the magnitude attribute. If ${\text{s }} > { }0$, probability-related evidence is accumulated before reward magnitude evidence, while if ${\text{s }} < { }0$, probability-related evidence is accumulated after reward magnitude evidence. The absolute value of ${\text{s}}$ gives the difference in starting times for the two attributes in seconds.

Model M2 differed from M1 in having one more attribute, which is previously accumulated reward, to affect the starting point of the evidence accumulation. Thus, M2 was specified such that the previously accumulated reward could shift the starting point toward acceptance or rejection of the choice as follows:

4-M2 $$E_{{\hat{0}}} = E_{0} + \omega_{A} V_{A}$$

where $E_{{\hat{0}}}$ indicates the shifted starting point by the previously accumulated reward. *ω*_*A*_ indicates the weighting strength (coefficient effect) given to the accumulated reward attribute and *V*_*A*_ represents the value of the thus far accumulated reward in the previous feedback trial. Note that the previously accumulated reward levels were transformed using a natural logarithm, and then z-scored across all participants and trials. Therefore, $\omega_{A}$ indicates the effect of a change of 1 standard deviation in the accumulated reward attribute on the starting point.

For the third (M3) model, we hypothesized that the previously accumulated reward would influence the drift rate parameter together with the reward probability and magnitude on the current trial. Specifically, because the information about the previously accumulated reward was given before each choice trial began, M3 assumed that the influence of the accumulated reward on evidence accumulation in the current choice trial was present from the onset of the choice until the participant made a decision. The reward probability and magnitude in the current trial could begin to be considered at different times, but neither could come before the previously accumulated reward. The drift rate parameter is therefore formulated as follows:

5-M3 $$\mu \left( t \right) = \left\{ {\begin{array}{*{20}l} {\omega_{A} V_{A} + \omega_{P} V_{P} } \hfill & { if\; {\text{s}} > 0 \wedge 0 < {\text{t}} < s} \hfill \\ {\omega_{A} V_{A} + \omega_{M} V_{M} } \hfill & {if\; s < 0 \wedge 0 < t < \left| s \right|} \hfill \\ {\omega_{A} V_{A} + \omega_{P} V_{P} + \omega_{M} V_{M} } \hfill & \quad \quad {if\; t > \left| s \right|} \hfill \\ \end{array} } \right.$$

$V_{A}$, $V_{P}$, and $V_{M}$ are the transformed values using the same methods in M2. However, the $\omega_{A}$ indicates the effect of a change of 1 standard deviation in the previously accumulated reward attribute on the drift rate. In addition, the relative time parameter, *s,* denotes the difference in starting times for the probability and magnitude attributes in seconds. Similar to the starting time parameter in M1, if ${\text{s }} > { }0$, probability information is considered before magnitude information, while if ${\text{s }} < { }0$, probability information starts to be considered after magnitude information.

Lastly, M4 allowed for the possibility that participants might sample decision evidence based on the reward probability and magnitude information in the current trial first and then sample evidence about the accumulated reward information, or vice versa. The reward probability and magnitude in the current trial start being considered at the same time, but the timing for the accumulated reward information can be earlier or later than the current trial information. The drift rate parameter in the fourth model is formulated as follows:

6-M4 $$\mu \left( t \right) = \left\{ {\begin{array}{*{20}l} {\omega_{P} V_{P} + \omega_{M} V_{M} } \hfill & {if\; {\text{s}} > 0 \wedge 0 < {\text{t}} < s} \hfill \\ {\omega_{A} V_{A} } \hfill & {if\; s < 0 \wedge 0 < t < \left| s \right|} \hfill \\ {\omega_{P} V_{P} + \omega_{M} V_{M} + \omega_{A} V_{A} } \hfill & \quad \quad {if\; t > \left| s \right|} \hfill \\ \end{array} } \right.$$

where *s* therein indicates the time step at which both reward probability and magnitude attributes enter the accumulation process relative to the previously accumulated reward attribute. If ${\text{s }} > { }0$, the current information (i.e., probability and magnitude) is considered before the previously feedback information (i.e., accumulated reward), while if ${\text{s }} < { }0$, the current information starts to be considered after the previously feedback information. Furthermore, we also compare fits of all four models with a standard DDM without including the starting time parameters, which is functionally equivalent to not allowing starting time to differ between decision attributes (see Table S6 in Supplementary information).

The stDDM was fit using hierarchical Bayesian Chain Monte Carlo (MCMC) methods with the R package Rjags to estimate the approximate value of each parameter. This approach allows for the simultaneous estimation of both group- and individual-level parameters and allows data from all participants to inform the parameter estimates, while still taking individual differences into account^36^. Specifically, the hierarchical Bayesian fitting approach we used specified that individual parameter estimates were drawn from 4 separate group-level parameter distributions (2 conditions × 2 age groups). The group-level priors for the weighting strengths of probability, reward magnitude, previously accumulated reward, and the relative-starting time parameter were drawn from Gaussian distributions with the mean = 0 and SD = 1. Note that probability, reward magnitude, and previously accumulated reward values were divided by their respective standard deviations before fitting the model. The priors for the boundary and non-decision time parameters were drawn from uniform distributions with the range from 0.0001 to 5 and from 0 to 10, respectively. The priors for the bias (starting point) parameter were drawn from a beta distribution with the shape parameter α and scale β = 2. Moreover, all priors for the individual parameter were drawn from gamma distributions with shape parameter α = 1 and scale parameter β = 0.1. For all the reported parameters, the estimates were based on 30,000 samples (3 independent chains of 10,000 samples each) after 50,000 initial burn-in samples for each chain. Analyses and results of parameter recovery tests for this version of the stDDM and the current data set can be found in the Supplementary Information section.

### Model validation and comparison

The DIC was computed by the formula as follows^37^:

$$DIC = \overline{D} + pD$$

where $\overline{D}$ indicates the mean deviance in the model fitting and pD is the estimate for the effective number of parameters in the model by compairing the difference between the posterior mean of the deviance and the deviance at the posterior means of the parameters of interest. We further computed the DIC for each model. In addition, we used cross validation to compute the RMSE in choice outcomes for each of the four models. First, half of the total trials (odd or even trials) in each condition were fit with the four models to obtain the parameter values. Next, the mean parameter values (*true parameters*) of each participant were used to fit a generative version of the model to simulate the other half of binary choices in the given choice trials that were not used in the model fitting, and this simulation was performed 100 times for each trial. The simulated choices across the 100 datasets were averaged as the choice probability within subject, and then we computed the RMSE between the true and simulated choices across all trials. Lastly, the mean and standard deviation of the RMSEs within each age group and each condition were computed as the indicators of cross-validated model performance.

Apart from the DIC and RMSE values, we also investigated whether the models could reproduce the age- and condition-related differences in vaule sensitivity that were reported in our prior study^21^. To this end, we fitted the empirical choice data by a logistic regression that models the EVs as: win probability *  magnitude + (1 − win probability) * (− magnitude). This logistic model was also applied in several earlier studies using a similar decision-making task without the decision-aid^4,5,20^. We examined the effects of age and condition by the slopes logistic regression analyses across the whole range of EVs between age groups and conditions. Results with a 2 × 2 non-parametric aligned rank ANOVA^38^ showed significant main effects on age (*p* < 0.05) and condition (*p* < 0.05). Younger adults showed a larger slope than older adults, and the slope values in both age groups increased in the decision-aid compared to the non-aid (control) condition. In addition, an age × condition interaction was also observed (*p* < 0.05), which was mainly driven by a larger increase in EV by decision-aid in younger relative to older adults. These procedures mirror the analyses that were applied in our prior study to examine age and condition effects. Altogether, these analyses resulted in three indicators: DIC, RMSE, and choice patterns for the model comparison. The best fitting model was determined by these three indicators and then used for further analyses.

### Comparison of parameter estimates between age group and condition

Effects of adult age differences and increased information saliency on each parameter of the best fitting model were tested by comparing the entire posterior distributions of the group-level hyperparameters (3 independent chains of 10,000 samples each of 2 age groups × 2 experimental conditions). The differences in the posterior distributions between the age groups (mean of the two conditions in younger—mean of the two conditions in older age group) or between the experimental conditions (mean of the two age groups in decision-aid—mean of the two age groups in control) were computed to indicate the main effects. The interaction for age group × condition was denoted by subtracting the aid-induced effects (decision-aid − control) in the older age group from the aid-induced effects in the younger one. Moreover, we computed the mean of the differences of the posterior distributions, 95% highest density interval (HDI), and posterior probability (PP) of a difference above 0 in the posterior distributions to examine the significance of the statistical comparisons. The HDI was applied using the “hdi” function of the package “HDInterval” in R.

## Supplementary Information

Supplementary Information.

## Data Availability

The OSF DOI link to the codes of model fitting and parameter recovery as well as the empirical and simulated data is: https://doi.org/10.17605/OSF.IO/K8G3.
