# Supplementary material for: Older adults process the probability of winning sooner but weigh it less during lottery decisions
Source: Sci Rep. 2022 Jul 5;12:11381. doi: 10.1038/s41598-022-15432-y (PMC9256676; doi:10.1038/s41598-022-15432-y)
Supplement: Supplementary file 1 — Supplementary Information. [file 41598_2022_15432_MOESM1_ESM.docx]

**Results of the simulated choice patterns in the M1, M2, M3, and M4 models.** An equivalent aligned rank test for a nonparametric analysis was applied on the simulated choice datasets generated from each model. The data from M3 replicated the results from the empirical data, specifically younger adults showed a higher slope (i.e., EV sensitivity) than older adults across both conditions (*p* < 0.05). The decision-aid increased the slopes in both age groups (*p* < 0.05), and the increased slope was larger in younger than in older adults (age group × condition: *p* < 0.05). None of the other three models generated behavior that matched the empirical results. In the M1 model, results showed a main effect on condition (*p* < 0.05) and interaction between age group and condition (*p* < 0.05) but no main effect on age group (*p* = 0.4). Results in the M2 model revealed a main effect on age group (*p* < 0.05) and interaction between age group and condition (*p* < 0.05) but no main effect on condition (*p* = 0.1). The M4 model showed no main effect on age group (*p* = 0.6) or condition (*p* = 0.5).

**Analysis and results of parameter recovery in the best fitting model (M3).** To assess whether the best fitting model (M3) is capable of reliably identifying the effects of adult age and increased information saliency in value-based decision-making, we ran a parameter recovery analysis. First, the mean parameter values (*true parameters*) of each participant were used to fit a generative version of the model to simulate the behavioral datasets. Next, we used our model-fitting procedure to fit the simulated behavioral datasets to obtain the estimated parameters. Fig. S1 and S2 (left panels) depict the posterior distributions of the hyperparameters for each parameter in the simulated data. To examine whether the effects of adult age and condition observed on the true parameters in the empirical data did not differ from those on the estimated parameters in the simulated data, we computed the mean of the differences of the posterior distributions, 95% highest density interval (HDI), and posterior probability (PP) of a difference above 0 in the posterior distributions on the main and interaction effects of age group and condition between the true and estimated parameters. Table S5 reports the differences of the main and interaction effects on the posterior distributions between the true and estimated parameters. Results showed that all main effects of adult age and condition and their interaction observed on the true parameters did not differ from those on the estimated parameters (Fig. S1 and S2, right panels). These findings indicate that the effects found on the true parameters of the best fitting model (M3) in the empirical data were accurate and reliable.

In addition, the correlation analyses between the true and estimated parameters at the individual level were also applied. Results showed that the correlation coefficients for the three weighting strengths for probability, reward magnitude and previously accumulated reward, non-decision time, boundary, and bias were around 0.8 in all age groups and all conditions (see Table S6). The correlation coefficients for the relative-starting time were approximately 0.7 in younger and older adults.

**Table S1.** Mean and highest density interval of the posterior distributions of the hyperparameters on each parameter in the M1 model.

| **Parameter**  **Group and Condition** | | **Probability**  **Coefficient** | **Magnitude**  **Coefficient** | **Probability minus Magnitude Coefficient** | **Relative-starting Time** | **Non-decision Time** | **Boundary**  **(Threshold)** | **Bias**  **(Starting Point)** |  |
| --- | --- | --- | --- | --- | --- | --- | --- | --- | --- |
| **YA** | **Control** | 1.99  [1.81 2.19] | 0.39  [-0.01 0.79] | 1.60  [1.15 2.03] | 0.69  [0.53 0.68] | 0.53  [0.49 0.57] | 2.32  [2.18 2.47] | 0.50  [0.47 0.54] |  |
|  | **Aid** | 2.47  [2.22 2.74] | 0.79  [0.27 1.35] | 1.68  [1.07 2.26] | 0.75  [0.60 0.91] | 0.49  [0.45 0.52] | 2.29  [2.16 2.42] | 0.51  [0.48 0.55] |  |
| **OA** | **Control** | 1.32  [1.16 1.46] | 0.02  [-0.07 0.11] | 1.30  [1.12 1.48] | 0.09  [0.04 0.14] | 0.70  [0.65 0.75] | 2.45  [2.31 2.58] | 0.51  [0.48 0.54] |  |
|  | **Aid** | 1.52  [1.36 1.69] | 0.21  [0.00 0.44] | 1.31  [1.03 1.59] | 0.58  [0.32 0.87] | 0.64  [0.59 0.69] | 2.54  [2.38 2.71] | 0.53  [0.50 0.56] |  |
| Information in the table reports the mean and 95% HDI in square brackets of the distribution of the posterior estimates for each M1 parameter. Abbreviations: HDI = highest density interval; YA = younger adults; OA = older adults; Aid = decision-aid. | | | | | | | | | |

**Table S2.** Mean and highest density interval of the posterior distributions of the hyperparameters on each parameter in the M2 model.

| **Parameter**  **Group and Condition** | | **Probability**  **Coefficient** | **Magnitude**  **Coefficient** | **Probability minus Magnitude Coefficient** | **Accumulated Reward Coefficient** | | | **Relative-starting Time** | **Non-decision Time** | **Boundary**  **(Threshold)** | **Bias**  **(Starting Point)** |
| --- | --- | --- | --- | --- | --- | --- | --- | --- | --- | --- | --- |
| **YA** | **Control** | 2.01  [1.82 2.20] | 0.36  [-0.04 0.76] | 1.64  [1.20 2.08] | | -0.14  [-0.20 -0.08] | 0.69  [0.54 0.86] | | 0.53  [0.49 0.57] | 2.33  [2.19 2.48] | 0.65  [0.59 0.70] |
|  | **Aid** | 2.48  [2.23 2.74] | 0.76  [0.22 1.30] | 1.72  [1.10 2.31] | | -0.14  [-0.20 -0.08] | 0.75  [0.60 0.91] | | 0.49  [0.45 0.53] | 2.29  [2.17 2.43] | 0.65  [0.60 0.70] |
| **OA** | **Control** | 1.32  [1.17 1.47] | 0.01  [-0.08 0.10] | 1.31  [1.13 1.48] | | -0.05  [-0.10 0.01] | 0.09  [0.04 0.14] | | 0.71  [0.66 0.76] | 2.45  [2.32 2.59] | 0.56  [0.51 0.60] |
|  | **Aid** | 1.54  [1.36 1.71] | 0.19  [-0.04 0.42] | 1.34  [1.05 1.62] | | -0.13  [-0.18 -0.08] | 0.57  [0.25 0.96] | | 0.64  [0.59 0.69] | 2.56  [2.39 2.73] | 0.65  [0.60 0.70] |
| Information in the table reports the mean and 95% HDI in square brackets of the distribution of the posterior estimates for each M2 parameter. Abbreviations: HDI = highest density interval; YA = younger adults; OA = older adults; Aid = decision-aid. | | | | | | | | | | | |

**Table S3.** Mean and highest density interval of the posterior distributions of the hyperparameters on each parameter in the M3 model.

| **Parameter**  **Group and Condition** | | **Probability**  **Coefficient** | **Magnitude**  **Coefficient** | **Probability minus Magnitude Coefficient** | **Accumulated Reward Coefficient** | | | **Relative-starting Time** | **Non-decision Time** | **Boundary**  **(Threshold)** | **Bias**  **(Starting Point)** |
| --- | --- | --- | --- | --- | --- | --- | --- | --- | --- | --- | --- |
| **YA** | **Control** | 2.02  [1.83 2.21] | 0.52  [0.18 0.86] | 1.50  [1.11 1.87] | | -0.18  [-0.31 -0.04] | 0.56  [0.40 0.72] | | 0.53  [0.49 0.56] | 2.34  [2.19 2.49] | 0.52  [0.49 0.55] |
|  | **Aid** | 2.50  [2.24 2.75] | 0.94  [0.49 1.39] | 1.56  [1.03 2.06] | | -0.23  [-0.37 -0.11] | 0.66  [0.49 0.83] | | 0.49  [0.45 0.52] | 2.30  [2.18 2.43] | 0.53  [0.50 0.57] |
| **OA** | **Control** | 1.35  [1.19 1.51] | 0.47  [0.17 0.79] | 0.88  [0.53 1.22] | | -0.12  [-0.26 0.04] | 1.04  [0.72 1.40] | | 0.70  [0.65 0.75] | 2.47  [2.33 2.61] | 0.52  [0.49 0.55] |
|  | **Aid** | 1.55  [1.38 1.72] | 0.46  [0.20 0.74] | 1.08  [0.76 1.39] | | -0.14  [-0.29 0.02] | 0.90  [0.57 1.25] | | 0.64  [0.59 0.68] | 2.56  [2.39 2.73] | 0.54  [0.51 0.57] |
| Information in the table reports the mean and 95% HDI in square brackets of the distribution of the posterior estimates for each M3 parameter. Abbreviations: HDI = highest density interval; YA = younger adults; OA = older adults; Aid = decision-aid. | | | | | | | | | | | |

**Table S4.** Mean and highest density interval of the posterior distributions of the hyperparameters on each parameter in the M4 model.

| **Parameter**  **Group and Condition** | | **Probability**  **Coefficient** | **Magnitude**  **Coefficient** | **Probability minus Magnitude Coefficient** | **Accumulated Reward Coefficient** | | | **Relative-starting Time** | **Non-decision Time** | **Boundary**  **(Threshold)** | **Bias**  **(Starting Point)** |
| --- | --- | --- | --- | --- | --- | --- | --- | --- | --- | --- | --- |
| **YA** | **Control** | 2.01  [1.83 2.20] | 0.12  [0.03 0.21] | 1.90  [1.69 2.10] | | 0.14  [-0.29 0.58] | -0.64  [-0.89 -0.42] | | 0.53  [0.49 0.56] | 2.33  [2.19 2.48] | 0.50  [0.47 0.54] |
|  | **Aid** | 2.47  [2.22 2.72] | 0.07  [-0.01 0.16] | 2.40  [2.13 2.66] | | 0.69  [-0.02 1.46] | -0.86  [-1.10 -0.61] | | 0.49  [0.45 0.52] | 2.29  [2.16 2.42] | 0.51  [0.48 0.55] |
| **OA** | **Control** | 1.34  [1.19 1.49] | 0.10  [0.03 0.17] | 1.24  [1.08 1.41] | | -0.15  [-0.30 0.00] | -0.09  [-0.13 -0.05] | | 0.70  [0.65 0.75] | 2.47  [2.33 2.61] | 0.52  [0.49 0.55] |
|  | **Aid** | 1.55  [1.38 1.73] | 0.05  [-0.03 0.12] | 1.51  [1.32 1.69] | | 0.17  [-0.14 0.49] | -0.59  [-0.89 -0.31] | | 0.64  [0.59 0.68] | 2.56  [2.39 2.73] | 0.53  [0.50 0.56] |
| Information in the table reports the mean and 95% HDI in square brackets of the distribution of the posterior estimates for each M4 parameter. Abbreviations: HDI = highest density interval; YA = younger adults; OA = older adults; Aid = decision-aid. | | | | | | | | | | | |

**Table S5.** Coefficient weights on probability, magnitude and accumulated reward level in the M3 model.

| **Parameter**  **Group and Condition** | | **Probability**  **Weight** | **Magnitude**  **Weight** | **Accumulated Reward Weight** | | |
| --- | --- | --- | --- | --- | --- | --- |
| **YA** | **Control** | 0.86 | 0.22 | -0.08 |  |  |
|  | **Aid** | 0.78 | 0.29 | -0.07 |  |  |
| **OA** | **Control** | 0.79 | 0.28 | -0.07 |  |  |
|  | **Aid** | 0.83 | 0.25 | -0.07 |  |  |
| Abbreviations: YA = younger adults; OA = older adults; Aid = decision-aid. Note. Coefficit weights were computed by dividing each drift coefficient by the sum of all coefficients for the three attributes. | | | | | |  |

**Table S6.** Deviance information criterion values of starting-time and standard drift diffusion models for the model comparisons.

| **Model**  **Group and Condition** | | **M1** | **M2** | **M3** | **M4** | **Standard**  **DDM** |
| --- | --- | --- | --- | --- | --- | --- |
| **YA** | **Control** | 5104.54 | 4991.09 | 4817.21 | 4890.84 | 4955.72 |
|  | **Aid** | 1000.20 | 914.21 | 798.73 | 967.99 | 778.24 |
| **OA** | **Control** | 15002.14 | 14953.44 | 14560.64 | 14613.66 | 14664.71 |
|  | **Aid** | 12978.38 | 12752.02 | 12572.21 | 12620.63 | 12603.75 |
| Note. Standard DDM did not include the time parameters, and thus synchronized starting times of all attributes in influencing the evidence accumulation. Abbreviations: YA = younger adults; OA = older adults; Aid = decision-aid. | | | | | | |

**Table S7.** Differences in the posterior distributions of the age and condition effects on each parameter of the best fitting model (M3) in the empirical compared to simulated data.

| **Parameter**  **Effect** | **Probability**  **Weight** | **Magnitude**  **Weight** | **Accumulated Reward Weight** | **Relative-starting Time** | **Non-decision Time** | **Boundary**  **(Threshold)** | **Bias**  **(Starting Point)** |
| --- | --- | --- | --- | --- | --- | --- | --- |
| **Age**  [(True: YA – OA) –  (Estimated: YA – OA)] | -0.06  [-0.34 0.22]  PP = 0.35 | -0.01  [-0.57 0.52]  PP = 0.48 | -0.01  [-0.21 0.18]  PP = 0.46 | 0.08  [-0.31 0.49]  PP = 0.66 | 0.02  [-0.04 0.08]  PP = 0.71 | -0.12  [-0.31 0.07]  PP = 0.11 | 0.01  [-0.04 0.05]  PP = 0.68 |
| **Condition**  [(True: Aid – Control) - (Estimated: Aid – Control)] | -0.03  [-0.31 0.25]  PP = 0.43 | -0.14  [-0.68 0.40]  PP = 0.31 | 0.00  [-0.19 0.19]  PP = 0.51 | -0.07  [-0.48 0.31]  PP = 0.36 | 0.00  [-0.06 0.06]  PP = 0.53 | -0.04  [-0.23 0.15]  PP = 0.35 | 0.01  [-0.04 0.05]  PP = 0.60 |
| **Age x Condition**  [True: (YA: Aid - Control) - (OA: Aid – Control)] - [Estimated: (YA: Aid - Control) - (OA: Aid – Control)] | -0.02  [-0.56 0.57]  PP = 0.47 | 0.34  [-0.75 1.42]  PP = 0.73 | -0.01  [-0.42 0.37]  PP = 0.48 | 0.32  [-0.46 1.14]  PP = 0.79 | 0.01  [-0.11 0.13]  PP = 0.54 | -0.05  [-0.43 0.33]  PP = 0.39 | 0.00  [-0.10 0.08]  PP = 0.46 |
| This table reports the mean and 95% HDI in square brackets of the distribution of the differences in posterior estimates for each stDDM parameter. The posterior probability showing that the difference is greater than zero is listed in parentheses. Abbreviations: HDI = highest density interval; PP = posterior probability; YA = younger adults; OA = older adults; Aid = decision-aid. | | | | | | | |

**Table S8.** Pearson’s correlation coefficients between the true and estimated stDDM (M3) parameters at the individual level.

| **Age Group** | **Condition** | **Weighting Strength of Probability** | **Weighting Strength of Magnitude** | **Weighting Strength of Accumulated Reward** | **Relative-starting Time** | **Non-decision Time** | **Boundary**  **(Threshold)** | **Bias**  **(Starting point)** |
| --- | --- | --- | --- | --- | --- | --- | --- | --- |
| YA | Control | 0.97 | 0.91 | 0.97 | 0.71 | 0.94 | 0.89 | 0.90 |
|  | Aid | 0.96 | 0.75 | 0.96 | 0.60 | 0.97 | 0.92 | 0.86 |
| OA | Control | 0.97 | 0.78 | 0.97 | 0.71 | 0.97 | 0.91 | 0.90 |
|  | Aid | 0.95 | 0.85 | 0.95 | 0.77 | 0.93 | 0.92 | 0.87 |

Note. YA = younger adults; OA = older adults; Aid= decision-aid.


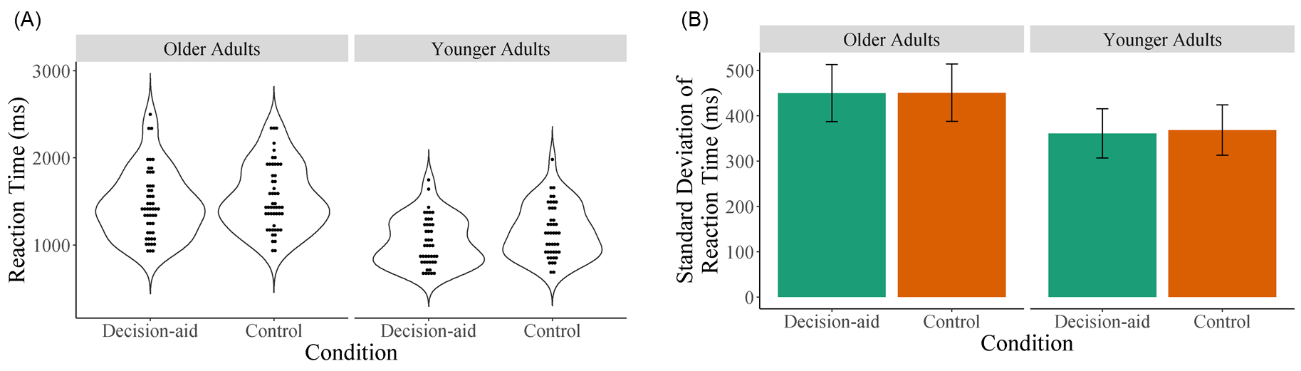


**Fig. S1. Reaction times in each age group and condition.** (A) Distributions of mean reaction times at individual level in younger and older adults. (B) Mean of reaction time variabilities (standard deviations) at group level in younger and older adults.


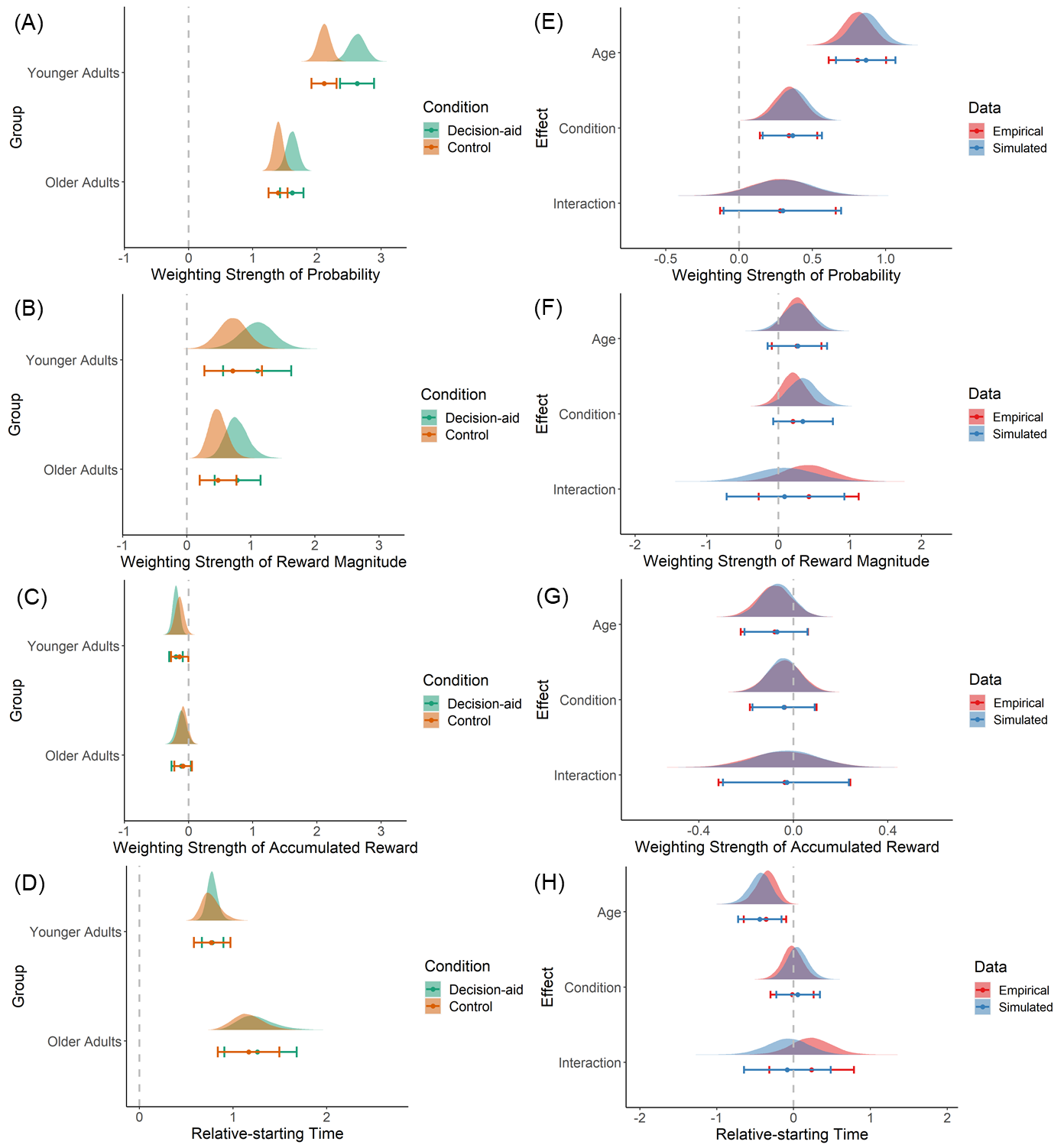


**Fig. S2. Effects of age group and condition on the estimated drift weight and relative starting time parameters of stDDM (M3).** (A-D) Posterior distributions of the estimated hyperparameters (group level in the simulated data) for the drift weights and relative starting times from the best fitting model (M3) in all experimental conditions in both age groups. (E-H) Differences of the posterior distributions on the main and interaction effects of age group and condition between the true and estimated drift weights and relative starting time parameter. Note. Dots and error bars shown below the distribution plots indicate the mean and 95% highest density interval, respectively.


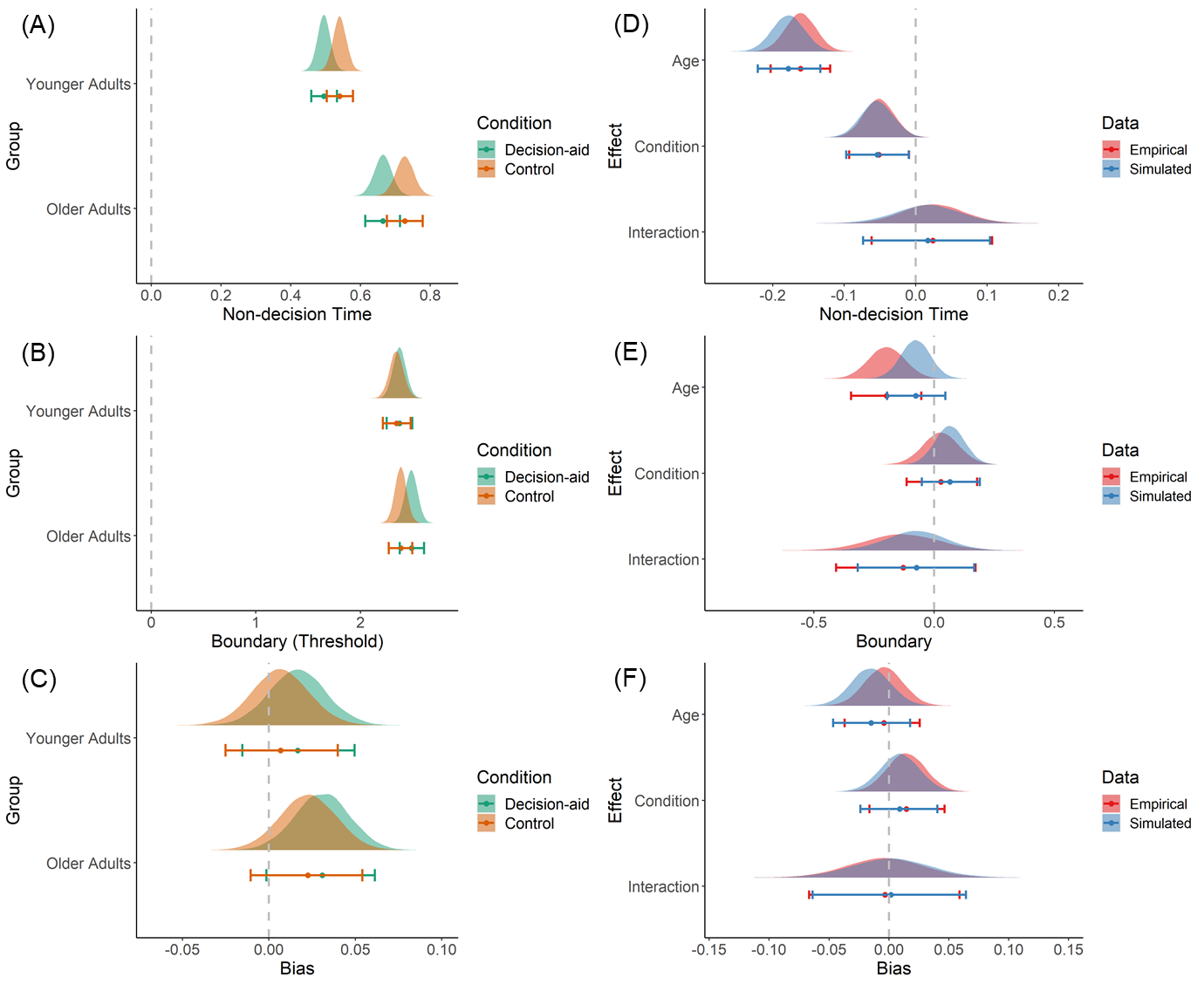


**Fig. S3. Effects of age group and condition on the estimated non-decision time, boundary, and starting point bias parameters of stDDM (M3).** (A-C) Posterior distributions of the estimated hyperparameters (group level in the simulated data) for non-decision times, boundary separation (threshold), and starting point bias from the best fitting model (M3) in all experimental conditions in both age groups. (D-F) Differences of the posterior distributions on the main and interaction effects of age group and condition between the true and estimated non-decision time, boundary (threshold), and bias (starting point) parameters. Note. Dots and error bars shown below the distribution plots indicate the mean and 95% highest density interval, respectively. The distributions of the bias parameter are shifted by the parameter values minus 0.5 to have the same reference point at zero as other parameters.
